# Potential of Stem-Cell-Induced Peripheral Nerve Regeneration: From Animal Models to Clinical Trials

**DOI:** 10.3390/life14121536

**Published:** 2024-11-23

**Authors:** Taylor M. Wynne, Virginia Grey Fritz, Zachary T. Simmons, Malek Zahed, Ananya Seth, Tamir Abbasi, Michael J. Reymundi, Kelly C. S. Roballo

**Affiliations:** 1 Department of Biomedical, Edward Via College of Osteopathic Medicine, Blacksburg, VA 24060, USA; twynne@vt.vcom.edu (T.M.W.); vfritz@vcom.edu (V.G.F.); zsimmons@vcom.edu (Z.T.S.); mzahed@vt.vcom.edu (M.Z.); aseth@vt.vcom.edu (A.S.); tabbasi@vt.vcom.edu (T.A.); mreymundipabon@vt.vcom.edu (M.J.R.); 2 Department of Biomedical Sciences and Pathobiology, Virginia Maryland College of Veterinary Medicine, Virginia Tech, Blacksburg, VA 24061, USA

**Keywords:** peripheral nervous system, peripheral nerve injury, stem cell, transplant, regeneration

## Abstract

Peripheral nerve injury has become an increasingly prevalent clinical concern, causing great morbidity in the community. Although there have been significant advancements in the treatment of peripheral nerve damage in recent years, the issue of long-term nerve regeneration remains. Furthermore, Wallerian degeneration has created an obstacle to long-term nerve regeneration. For this reason, there has been extensive research on the use of exogenous and endogenous stem cells as an adjunct or even primary treatment option for peripheral nerve injury. The plasticity and inducibility of stem cells make them an enticing option for initiating neuronal cell regrowth and optimal sensory and functional nerve regeneration. Peripheral nerve injury has a broad range of causative factors and etiologies. As such, unique stem cell-induced peripheral nerve treatments are being investigated to ameliorate the damage incited by all causes, including trauma, neuropathy, and systemic neurodegenerative diseases. This review is oriented to outline the potential role of stem cell therapies in peripheral nerve injury versus the current standards of care, compare the benefits and drawbacks of specific stem cell lines under investigation, and highlight the current models of stem cell therapy in the peripheral nervous system, with the ultimate goal of narrowing down and optimizing the role and scope of stem cell therapy in peripheral nerve injury.

## 1. Introduction

The peripheral nervous system (PNS) links the internal and external environment to the central nervous system (CNS). The PNS is composed of a multitude of voluntary sensory and motor nerves along with involuntary, autonomic nervous system components. The nerves are highly organized structures that make a bridge between the PNS and CNS [1]. Nerves, also known as axons, are also the signaling structures of neurons. Their electrolyte channels transmit electrical signals from upstream neurons, sensory receptors, and neurotransmitters. Some of these axons are insulated by myelin, a polypeptide structure produced by Schwann cells, the supporting glial cells of the peripheral nervous system [1].

Peripheral nerve injury (PNI), especially from trauma, varies in location, severity, prognosis, and treatment [2]. An epidemiological analysis of 5026 peripheral nerve lesions at a European Level I Trauma center from 2012 to 2020 found that 88.3% of lesions were in the upper body extremity. Additionally, 52.2% of all peripheral nerve lesions seen were the result of an acute traumatic injury, while the remaining 47.8% of lesions were the result of a non-traumatic cause such as compression, tumor growth, or neuropathy [2,3,4,5].

Stem cells are a cell type that can self-renew and differentiate into specific cell types [6]. There are several types of stem cells: embryonic stem cells and adult stem cells. Embryonic stem cells (ESCs) have the ability to differentiate in all the cell types of the adult organism. Adult stem cells have limited ability to differentiate; however, they are essential for replenishing cells that are aging or damaged in an adult organism [7,8]. The therapeutic potential of stem cells lies in adult stem cells, which can be used from autologous or allogeneic sources. Some examples of sources of allogenic stem cells include bone marrow (hematopoietic stem cells—HSCs), amniotic tissue (amnion-derived stem cells), adipose tissue (adipose-derived stem cells—ADSCs) [9], skeletal muscle-derived stem cells (SKM-SCs), and genetically modified cells such as induced pluripotent stem cells (iPSCs).

## 2. Discussion

### 2.1. Current Intrinsic Peripheral Nerve Repair Mechanisms Targeted in Stem Cell Treatments

Schwann cells are a type of glial cell in the PNS that act as support cells to the peripheral nerves. They play a role in immune response, myelination, and axonal growth. PNIs are generally categorized into two types: axonotmesis, which is characterized by the disruption of axons but a sparing of the connective tissues and Schwann cells, and neurotmesis, where all three of these components are interrupted. These two forms of peripheral nerve injuries are related to crush/stretch and transection injuries, respectively. In the latter, the initial inflammatory response builds a connective tissue bridge that spans the two transected stumps, which forms a scaffolding for immune cells and growth factors to build upon [10]. Irrespective of which injury occurs, the axons distal to the injury undergo Wallerian degeneration, leading to a clear pathway for the proximal axons, still supported by their cell body, to survive, grow, and replace the lost axons. If axonal regrowth is not possible, collateral sprouting can support the injured nerves via new innervations [10,11].

A major component of the regenerative process is that Schwann cells (SCs) can change their genetic expression to revert and de-differentiate into a more primitive cell line specialized in the regeneration and transcription of neurotrophic and growth factors [10,11,12]. SCs can take the form of three main phenotypes: myelin-producing Schwann cells, non-myelin-producing Remak cells, and scaffolding-associated Büngner Schwann cells [11]. This quality is unique to the peripheral nervous system and the reason why peripheral neurons can recover in contrast to the central nervous system and its glial cells. Büngner Schwann cells proliferate distal to the injury site and form bands that act as guides to newly sprouting axons, while Remak cells are specialized in axonal regeneration, support, and repair in the proximal stump [11,13]. Yet, even with these adaptations and the most ideal circumstances, axons are said to grow 1–4 mm/day in humans [10,13]. At this rate, distal organ and tissue atrophy may lead to permanent loss of function long before the sprouting axons reach the de-innervated tissue. This is exacerbated by the waning of the immune and regenerative response over time and the loss of guidance to the novel axonal sprouts [13].

**Figure 1:**
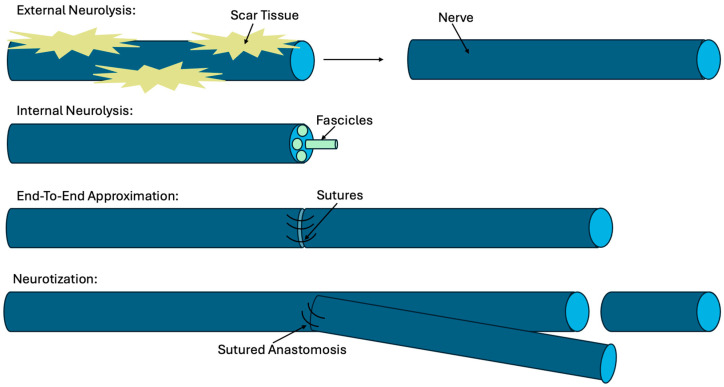
Diagram depicting the various modalities of peripheral nerve repair following injury.

There are multiple modalities and techniques that attempt to support and accelerate this process in peripheral nerve injuries. Below, some of these modalities are depicted and listed (Figure 1):

- Neurolysis seeks to isolate injured nerves from surrounding scar tissue to promote more efficient healing. This technique can be external or internal. External neurolysis dissects the nerve from surrounding structures, while internal neurolysis isolates intraneuronal fascicles, which carry the conducting axons within [10].
- End-to-end approximation involves suturing and reattaching transected nerves. If the gap is too great, then nerve grafting can be achieved via the harvest of autologous nerve grafts from peripheral sites, most commonly the sural nerve, which remains a gold standard treatment [10]. However, nerve grafts are not suitable for mixed (sensory and motor) nerves nor for grafting a sensory nerve into a motor nerve. An alternate solution to such scenarios is nerve conduits such as autologous veins or synthetic collagen conduits [13]. All these forms of repair have shown similar success rates ranging from 40 to 70% that vary depending on the extent of damage and the length of transection [1,13].
- Neurotization, or nerve transfer, uses an intact local nerve that is anastomosed to the distal axons of the damaged nerve, essentially bypassing the injury site when there are graft length limitations [10].

Yet, with all these forms of repair, the main regenerative process cannot be bypassed, and these different modalities simply act as support for this essential mechanism. The literature has shown that when Schwann cells are spared, as in crush injuries, nerve regeneration is quick, efficient, and successful in restoring function [12]. However, when nerves are transected and Schwann cells are disrupted, regeneration is variable and most often does not achieve full function, even with repair.

It has been well-established that there is only a brief therapeutic window of 18 months available to establish optimal nerve regeneration following peripheral nerve injury, making efficient diagnosis and treatment initiation essential [14]. Therefore, novel studies are exploring the role of stem cells and stem cell therapy in assisting the natural process of regeneration as a stand-alone therapy as well as an adjunctive method to surgical repair [15,16,17]. The goal of stem cell therapy is to maintain the immune and regenerative response, which has shown promising results with mesenchymal stem cells (MSCs) in animal models. MSCs have the ability to immunomodulate the injury site and other resident cells. Studies have shown that MSCs can convert to Schwann-like cells that express GFAP and neuron-like cells [9,10,17,18]. Furthermore, MSCs also upregulate immune cells, cytokines, and growth factors, which promote repair [9].

### 2.2. Current Clinical Application of Autologous Versus Allogeneic Stem Cell Therapy

To discuss the clinical implications of stem cells in peripheral nerve regeneration, we must have clear definitions of some of the types of stem cell derivations used. The most relevant to discuss here include autologous (transplant of stem cells from the host), allogeneic (transplant of stem cells from the same species), and xenogeneic (transplant of stem cells from another species) [19]. The source of stem cell origination in these categories varies from multipotent hematopoietic stem cells (HSCs), bone-marrow-derived stem cells (BMDSCs), adipose-derived stem cells (ADSCs), skeletal-muscle-derived stem cells (SKM-SCs), and many more as discussed throughout the paper. The harvesting procedures of these stem cells are variant as well, considering their source. For example, in humans, we commonly use multipotent hematopoietic stem cells (HSCs) for the treatment protocol in leukemia and lymphoma patients [20]. If they are derived from bone marrow, then they are also known as BMDSCs. These are typically harvested for autologous use by which an adult person will undergo pre-procedure blood testing to look for any hematologic abnormalities, cardiac testing to ensure the heart can sustain the procedure, and pulmonary testing to make sure the lungs can sustain the procedure, and then get put under general anesthesia. After proper surgical scrubbing and sanitizing have occurred by the medical team, the patient will be put in a prone position, and a special needle will be placed through the skin into the hip bone marrow cavity. Here, the aspiration of the desired stem cell occurs. The marrow is collected, filtered, and stored according to typical protocols not mentioned here. Surprisingly, this is one of the simpler methods of stem cell harvesting. These cells can also be isolated through blood filtering, but these stem cells will typically be less in number as compared to those of direct bone marrow aspirates since, at this point in their life cycle, they tend to differentiate and specify themselves [21]. These stem cell types, along with various others (ADSCs, MSCs, etc.), can be frozen and mixed with a preservative at or below a storage temperature indefinitely. This is typically done in case the patient may need more stem cells, but this applies to how we store them for allogeneic use as well. This information is listed to understand the importance of harvesting the stem cell types from whatever source they come from because each stem cell type is harvested in a specific way [21,22].

Of much importance, when discussing and performing any allogeneic transplant, the immune response in the host must be reduced to ensure successful transplantation. To reduce this response, human leukocyte antigen (HLA) testing must be performed, and immune system activity will need to be reduced, most commonly by pre-procedural steroids. All cells have HLA I or II markers, including most stem cells, and these polymorphic proteins vary drastically in their classification. When appropriately matching these HLA subtypes of the donor and recipient, it is vital to maximize acceptance from the host without immune response in any transplant type. Luckily, HLA matching is a common practice performed in all transplant procedures today, and peripheral nervous system transplants are known for their lack of fatal immune responses. This is due to the peripheral axon lacking the aforementioned HLA subtypes, specifically HLA I, showing much promise in future transplantation with any type of source, including allogeneic, autologous, and xenogeneic ones [23]. Despite the blunted immune response compared to other solid organ and cellular transplants, recent studies on the long-term survival of transplanted induced pluripotent stem-cell-derived neurospheres into rat sciatic nerves have shown that non-immunosuppressed allogenic transplants only survived 14 days post-transplant, while immunosuppressed allografts survived for as long as 365 days in the rats [24]. Nonetheless, these findings suggest a benefit for immunosuppressive therapy as an adjunct to stem cell-induced peripheral nerve regeneration therapy despite the observed limited immune response and rejection. The clinical significance when comparing the sources of stem cells will be discussed after the discussion of the animal models mentioned later on in this review.

### 2.3. Comparison Between Different Cell Types as Sources for Stem Cells with Advantages and Disadvantages in Relation to Peripheral Nerve Regeneration

Due to the stem cell’s ability to differentiate into many different cell types, the induction of specific cells allows for the development of a favorable microenvironment that promotes the proliferation of myelin and peripheral nerve regeneration. There are currently numerous stem cell sources being investigated for use in regenerative efforts for peripheral nerve injury. The various stem cell types available for therapeutic use and their advantages and disadvantages are identified in Table 1 and Table 2, demonstrating some possible applications of these cells at the pre-clinical level.

Induced pluripotent stem cells (iPSCs) can be extracted easily from an individual’s tissues, such as skin cells, which are then reprogrammed into stem cells with the ability to differentiate into a multitude of other cells. This characteristic not only allows for an infinite number of cells to be proliferated and a large source of availability but also an individualized potential treatment option for future patients. Despite the individualized benefit, the epigenetic memory of these induced cells is an important consideration as it may play a large factor in the tumorigenicity of iPSCs [25].

Human embryogenic stem cells (hESCs), derived from human blastocysts, are pluripotent cells that can be cultured and proliferate for long periods with the retained ability to differentiate [38]. While the differentiation of hESCs into somatic stem-cell-like precursors illustrates a promising conduit for peripheral nerve injury regeneration, the presence of undifferentiated hESCs may propagate the formation of teratomas and teratocarcinomas [30]. In addition, ethical considerations must be made when suggesting the use of hESCs.

Hair follicle pluripotent stem cells (HFPSs) provide an alternative for the tumorigenicity seen in hESCs and iPSCs. A benefit of using HFPSs also includes the lack of genetic manipulation necessary to adapt the cells to each prospective patient [28]. While there is an abundance of sources for HFPSs, the isolation process of the hair follicle pluripotent stem cell area is difficult and time-consuming, taking up to four weeks to procure nestin-positive HFPS cells [29].

The use of liposuction provides an effective and easy isolation of ADSCs as a potential source. These multipotent cells have been shown to differentiate into Schwann-cell-like cells when in a co-culture system with Schwann cells [8,18]. Another study provided insight into the release of humoral factors, Neu-1, and VEGFA as major factors in Schwann cell proliferation and migration, furthermore contributing to the promotion of peripheral nerve regeneration [33]. While the use of ADSCs shows promising advantages, a particular roadblock is seen with the removal of the differentiation medium, leading to a reversion back to stem cell morphology as a drop in humoral factor expression [32].

Similar to ADSCs, bone-marrow-derived stem cells (BMDSCs) co-cultured with SCs have shown a propensity to increase peripheral nerve injury regeneration comparable to autograft groups via differentiation into Schwann-cell-like cells [7,8]. However, comparing ADSCs and BMDSCs shows a significant difference in proliferation and differentiation to Schwann-cell-like cells with a preference for ADSCs [8]. Despite comparability in the use of ADSCs and BMDSCs, obtaining BMDSCs involves a painful and invasive extraction process, with lower cell yields leading to a lack of appeal for this method [8].

Human dental pulp stem cells (hDPSCs) are easily able to be harvested from wisdom teeth, minimizing the necessity for invasive procedures of extraction, which are common in other cell sources. hDPSCs’ expression in a multitude of neural cell markers indicates an ability to differentiate into neural lineages such as Schwann-cell-like cells as well as provide neuroprotective and neurotrophic effects when differentiated as compared to undifferentiated ones [35]. A recent research study performed by Sanen et al. confirmed that hDPSCs have increased angiogenic properties, allowing for greater endothelial migration and tube formation after differentiation into Schwann-like cells [36]. A setback for the use of hDPSCs would be storage demands at extremely low temperatures, requiring the use of a stem cell bank for cryopreservation [35].

While there has been a significant number of studies performed on the potential therapeutic effects of mouse skeletal-muscle-derived multipotent stem cells (mSKMDSCs), there is a need for more research on skeletal-muscle-derived stem cells (SKMDSCs). Tamaki et al. were able to demonstrate that SKMCSs have sustained multipotent differentiation abilities that allow for the formation of both skeletal myogenic lineages and peripheral-nerve-blood vessel lineages, proposing a potential source for stem cell sources [34].

**Table 2:** Pre-clinical application of various stem cell therapy trials in peripheral nerve regeneration (iPSCs: induced pluripotent stem cells, hfSCs: hair follicle stem cells, ESCs: embryogenic stem cells, ADSCs: adipose-derived stem cells, BMDSCs: bone-marrow-derived stem cells, SKMDSCs: skeletal-muscle-derived stem cells, hDPSCs: human dental pulp stem cells).

Cell Type	Cited Study	Model Species	Method of Stem Cell Delivery (Local or Systemic)	Peripheral Nerve Targeted	Results
iPSCs	[39]	Mouse	Local; iPSCs suspended in conduit, conduit then implanted directly into sciatic nerve	Sciatic nerve	At 4-, 8-, and 12-weeks post iPSC implantation, motor and sensory recovery was significantly improved along with significantly more abundant axonal regeneration in IPSC-treated mice as compared to controls.
	[40]	Rat	Local; iPSCs suspended in nerve conduit, conduit then implanted directly into sciatic nerve	Sciatic nerve	iPSCs differentiated into neural cells, specifically Schwann cells that accelerated myelination and regeneration at 1-month post-conduit implant, as compared to controls. There was also no evidence of teratoma formation at 1-year post-transplantation.
	[41]	Mouse	Local; iPSCs suspended in nerve conduit, conduit then implanted directly into sciatic nerve	Sciatic nerve	Addition of iPSCs to nerve conduit along with basic fibroblast growth factor hastened axon regrowth and quickened axon recovery.
HFSCs	[42]	Rat	Local; injected directly into area surrounding sciatic nerve lesion	Sciatic nerve	Greater sciatic nerve epineurium repair seen in HFSC-treated group, as compared to other experimental groups (*p* < 0.05).
	[43]	Mouse	Local; injected directly into severed sciatic nerve	Sciatic nerve	HFSCs differentiated into glial cells, promoting myelination and functional nerve regeneration.
ESCs	[44]	Rat	Tubular conduits with fibrin matrix filled with ESC-derived neural crest cells were inserted surgically	Sciatic nerve	NCCs in grafts produced factors that promoted nerve regeneration.
	[45]	Mice	Fibrin sealant and FGF-2 genetically modified hESCs were added surgically to the site of injury	Sciatic nerve	Using immunohistochemistry and the von Frey test, it was observed that the ipsilateral paw had increased sensory function and reflexes.
ADSCs	[46]	Sheep	ADSC-cellularized autograft	Peroneal nerve	Gait analysis was performed 12 m post-surgery, and the gait scale score was higher than that of the untreated group and the same for the decellularized autograft group.
	[47]	Rat	CRISPR-engineered-ADSC sheets wrapped around injury site	Sciatic nerve	CRISPR-ADSC sheets along with BV vector enhanced axonal regeneration, Schwann cell migration, and functional recovery.
BMDSCs	[48]	Dog	Local; injected directly into vein conduit immediately after nerve injury	Facial Nerve	The dogs treated with BM-MSC had significant improvements in lower eyelid, ear, life, and tongue function after 4 weeks compared to other groups. Grossly, the facial nerve graft of the dogs injected with BM-MSC showed significantly less adhesion scores compared to others. The facial nerves with BM-MSCs had more normal axons compared to those of other groups.
SKMSCs	[49]	Mouse	Local; injected immediately into the bridged collagen tube connecting both transected stumps. Local; needle injection through the skin and buttocks at one shot/week for 6 weeks for infusion around/inside the collagen tube.	Sciatic nerve	The nerve showed significant recovery of the reconnected number of axon and myelinated fibers as well as improved tetanic tension output measured by electrical stimulation.
hDPSCs	[50]	Rat	Local; human dental-pulp-derived stem cells and differentiated neuronal cells from DPSCs (DF-DPSCs) were transplanted along with fibrin glue scaffold and collaged tubulization into the sciatic nerve resection	Sciatic nerve	Both the DPSC and DF-DPSC groups showed increased behavioral activities and higher muscle contraction force at 12 weeks compared to those of the control groups. Pre-transplanted labeled PKH26 tracking dye showed transplanted cells differentiated into nerve cells. No difference was shown in nerve regeneration between DPSC groups and DF-DPSC-transplanted groups.

### 2.4. Optimization of Protocols for Neuronal Stem Cell Differentiation in Peripheral Nerve Regeneration

**Figure 2:**
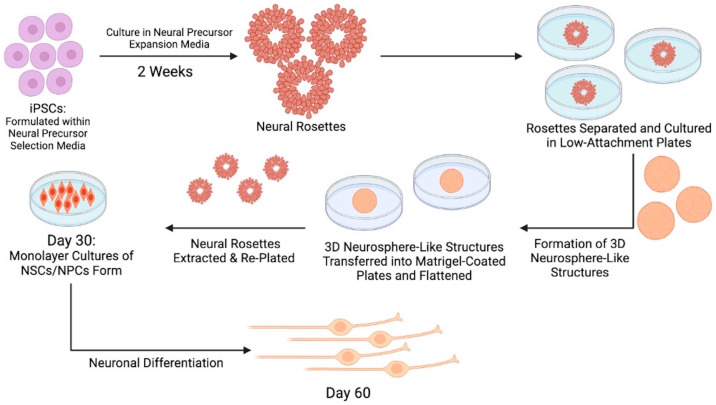
Schematic flow diagram outlining cell culture protocol steps to differentiate induced pluripotent stem cells (iPSCs) into fully differentiated neuronal cell types. Made using BioRender.com.

Despite the need for additional pre-clinical and clinical testing to better solidify the most optimal source of stem cells for peripheral nerve damage, it is clear that the potential use of stem cells in peripheral nerve regeneration shows great clinical promise [51]. Regardless of the stem cell source, inducing stem cell differentiation into various neuronal cell types is a delicate and time-consuming process [26]. Each neuronal stem cell line has a unique differentiation protocol, many of which are still under investigation for optimization at this time. In one commonly replicated cell culture protocol starting with induced pluripotent stem cells (iPSCs), the process requires stem cell culture within a formulated neural precursor selection medium, followed by culture in a neural precursor expansion medium for a total of 2 weeks to form neural rosettes. At this stage, the cell conglomerates, termed neural rosettes, will begin to express neuronal stem cell markers, and the rosettes are then divided and cultured into multiple, separate low-attachment plates where they begin to form 3D neurosphere-like structures containing aggregates of neural rosettes. Then, the neurosphere-like structures are transferred into Matrigel-coated plates, flattened, and the neural rosettes are extracted. Finally, the rosettes are re-plated to form new, purified neurosphere-like structures that are eventually used to develop monolayer cultures of neural stem cells (NSCs) and early neuronal progenitor cells (eNPCs). This whole process from iPSC to NSC/eNPC takes around 30 days and can take up to 60 days for complete neuronal differentiation [52]. Once the desired quantity of stem cells has been isolated, the cells can be directly extracted and used in treatments, or they must be preserved to retain their function [37]. The complete stem cell culture process is visually depicted in Figure 2.

Interestingly, stem cells of different embryonic origins have been shown to exhibit varying levels of survivability with the current gold standard methods for cryopreservation, and thus, ongoing research is set at the aim of developing new cryopreservation formulas to improve universal stem cell viability post-thawing [37,53]. Once transplanted, it is also vital to measure the viability of the stem cells in vivo. Current practice typically involves labeling the stem cells directly with either fluorophores, radioisotopes, or nanoparticles during cell culturing. Therefore, following an initial successful transplantation, the survival of these stem cells can be tracked through the small molecules with which they are labeled. Depending on the type of small molecule used for labeling, fluorescence imaging, nuclear imaging, or even magnetic resonance imaging can be used to analyze short and long-term stem cell viability [54].

### 2.5. Delivery Methods of Stem Cells into Peripheral Nerves

There are currently a multitude of potential delivery options under investigation for the use of stem cells as regenerative therapeutics in peripheral nerve injury [26]. It is known that stem cells in the proper media can be directly injected into nerve endings to promote nerve regrowth [55]. Additionally, stem cells can be added to the matrix of natural nerve or vascular conduits with or without additional extracellular matrix components such as collagen and fibrin to help direct stem cell differentiation and encourage successful functional graft peripheral nerve transplant [56,57].

Most recently, advancements in 3D printing and personalized medicine have led to the creation of 3D-printed nerve guidance channels that can be injected with suspended stem cells and will ideally recreate and mimic the exact path and function of a patient’s peripheral nerve before injury [58]. In terms of stem cell “dosing” in peripheral nerve injuries, there is no current consensus on the ideal number of stem cells required for optimized therapeutic outcomes. One review reports that the stem cell count used in experiments ranges from as low as 4 × 10^3^ cells to as high as 2 × 10^7^ cells delivered in transplants [59]. Thus, the exact quantification and concentration of stem cells need to be further elucidated in future studies to enable a clinical correlation. Although many of these delivery vehicles are still in the preclinical stages, the advancements in stem cell research and regenerative medicine in recent years have made stem-cell-based tissue-engineered nerve grafts a very realistic treatment in the near future [60].

### 2.6. Stem Cell Transplantation in Animal Models of Peripheral Nerve Injury

MSCs used in animal model experiments are gathered from bone marrow, adipose tissue, amniotic fluid, and the umbilical cord [16], targeting the differentiation potential into SCs. After the stem cells are harvested, they must undergo an induction to differentiate into SCs. Then, the newly differentiated SCs will lead to axonal regeneration along the nerve extracellular matrix or structure. Many research projects have focused on how different tissue-derived stem cells perform in animal models related to stem cell differentiation, nerve function, and nerve histology, with the idea that stem cell transplantation will improve PNI outcomes.

In 2018, in a study conducted by Carnevale et al., undifferentiated hDPSCs were used to promote nerve regeneration and functional recovery of the sciatic nerve in a rat model. After the induction of the undifferentiated hDPSCs, the research demonstrated that it took two to three weeks for the stem cells to contain markers for SCs, such as GFAP and S100b. The stem cells showed the ability to differentiate into SC-like cells, but another important component of PNI research is how well the stem cells act as SCs to regenerate the structure of the nerve and lead to functional recovery. In the rat model, the rats were observed four weeks after injury, and the scientists performed a paw analysis for which they found the functional recovery was significant for the hDPSC-collagen scaffold transplant group compared to the transplanted collagen scaffold group and untreated injured group. A histological analysis was performed on the rat sciatic nerves afterward, and the 6 mm gap had significant nerve regeneration from the proximal to distal end that aligned with the nerve fibers. When compared to the uninjured control rats, the hDPSC nerve thickness resembled the control. Conversely, the collagen scaffold-only group only showed some nerve regeneration at the proximal end and discontinuous nerve regeneration without the alignment of nerve fibers [16]. Taking a human model into consideration, it is compelling to explore the length of time it would take for the human nerve to regenerate using this method. In the experiment, the injury was 6 mm, which is significant for a rat, but in a human model, nerve injury relating to neuropathy or trauma can be substantial, and the human sciatic nerve is sizable compared to that in the rat model, so it begs the question whether the outcomes of hDPSCs would be as promising in a larger model.

The research study performed by Tamez-Mata et al. in 2022 utilized adipose-derived stem cells (ADSCs) to observe the long-term outcomes of a PNI of the peroneal nerve in a sheep model. A 30 mm gap was excised from the peritoneal nerve in fourteen sheep. Seven sheep received an autograft, and the other seven received a decellularized ADSCs autograft. Within five hours, the ADSCs differentiated into Schwann cells containing the S100 marker. Compared to the rat model, the stem cells in this experiment took significantly less time to differentiate into SCs. The functional outcome was measured through gait analysis performed at three, six, and twelve months. At twelve months, the gait scale score was the same for the autograph and ADSCs groups and higher than the untreated control group. If the sheep were studied for a longer period, it would be interesting to see if the autograft and the recellularized graft would continue to show similar increased functionality or if one of the grafts would plateau at a certain gait scale number while the other would continue to show gait improvement. In the recellularized autograph group, there was a proximal-to-distal organization of the nerve fibers, but the recellularized graft showed a statistically significant lower organization score [46]. To further this research, researchers could analyze whether different tissue-derived stem cells provide better outcomes, as the hDPSCs in the previous study demonstrated marked organization of the fibers, and this study has significantly less organization. It is difficult to determine whether the decreased organization of fibers could have occurred due to the stem cell type or if it was because this was a larger animal model. The authors did not give a probable explanation for the disorganization of the nerve fibers.

Another experiment led by Daradka et al. used a saphenous vein autograft conduit and an injection of BMSCs to measure the healing outcomes of a facial nerve injury in a dog model. The study consisted of four groups of untreated, conduit-only, platelet-rich plasma-injected conduits and BMSC-injected conduits. The dogs’ facial nerve injuries and functions were studied for 8 weeks. The results showed that by the fourth week, there was an improvement in the position of the dogs’ ears, lower eyelids, tongue position, upper lip deviation, and amount of salivation. The BMSC-injected test group also showed less fibrosis and adhesions while demonstrating more revascularization compared to the other test groups [48].

Notably, all of these animal model research studies included a procedure where a surgeon meticulously excised a portion of the nerve out of a previously healthy animal, and then the surgeon replaced the section immediately with the graft. With a PNI, a patient could live with the nerve injury chronically, so research should be conducted to demonstrate if this method of stem cell transplant would apply to a patient with chronic nerve injury and/or other comorbidities.

Although humans are more complicated than the research animal models, the animal models within the preclinical stage help researchers gain insight into the risks, benefits, and possible complications of stem cell transplant that could arise in a human model. Since pre-clinical research is ongoing, it is compelling to see how the various animal models demonstrate the efficacy of stem cell transplant, how the animal model translates to the human model, and which animal representation resembles the closest to the human model once clinical trials begin.

Returning to our primary discussion of transplant options (autologous, allogeneic, or xenogeneic) for stem cells in peripheral nerve regeneration, the most commonly used cell types in any stem cell therapy are autologous (self)-BMDSCs. These stem cells have the potential to differentiate into glial-like cells, more specifically Schwann-like cells, and boost the regenerative capacity of peripheral nerves, as do many other stem cell types. A 2021 experiment led by Daradka et al. used autologous BMDSCs throughout their in vivo experiment [48]. Clinically, in on-site human use, this would be the most beneficial as we would be able to harvest the BMSCs from the patient with the PNI and use them as they showed in the mentioned experiment. The 2022 research study performed by Tamez-Mata et al. was an allogeneic study and showed just as much, if not more promise than the BMSC study due to demonstrating the longevity of the transplant over 12 months, and exhibiting this method was useful in a larger animal model [46]. Regarding xenogeneic transplants, a 2018 study conducted by Carnevale et al., with undifferentiated hDPSCs and the sciatic nerve in a rat model, showed just as much promise as the autologous and allogeneic studies. The hDPSCs allowed for not only nerve regeneration along the whole segment but also showed nerve fiber organization, which was lacking in both the autologous animal model studies [16]. All three sources of stem cells can be used, as seen above, but considering the availability of xenogeneic stem cells, this has the greatest potential in future research regarding peripheral nerve repairs with stem cell implications.

Rat models are used every day due to their reproductive rates, similarities in genetics to humans, and similarities in social activity to humans. If we combine the lack of fatal immune response, as mentioned in the Carnevale study, with the availability of rat stem cells, we expect useful stem cell therapy with rat models in the future of peripheral nerve repair. However, autologous and allogeneic therapies are highly desired due to the stem cells coming from the same animal and species, respectively [16]. Ongoing research has revealed that there is a difference between the fascicles of different nerves found between men and women, so it begs the question of whether there are different nerve fascicle numbers and organizations between humans and various animal models, which would affect the healing and outcomes of PNI surgery. In this case, it would make sense to analyze whether human-derived stem cells would perform better in humans because those stem cells are originally destined to become human neuronal functional cells, which will form the organization and structure found in human nerve fascicles.

### 2.7. Clinical Trials Investigating the Efficacy of Stem Cell Therapy for Peripheral Nerve Repair in Human Subjects

SC therapy is a growing field and has been used as a therapeutic in different types of injuries, proving its use amongst a multitude of animal models. However, the use of SCs in peripheral nerve injuries remains novel, and further research needs to be performed to test its effect as a therapeutic option in human peripheral nerve injuries. As it currently stands, there are a significant number of new and/or ongoing human clinical trials looking into the utility and efficacy of stem cell therapy for various peripheral nerve injuries. Despite the ongoing nature of many of these studies, their initial results have been very promising for the clinical future of stem-cell-induced peripheral nerve regeneration and are summarized in Table 3.

## 3. Conclusions

The accumulation of research, mainly in in vitro and in vivo animal models at present, supports the effectiveness of stem cell therapy as a potential therapeutic method in different types of peripheral nerve injuries; however, there is currently a large array of ongoing clinical trials to further explore the efficacy and outcomes of stem-cell-induced peripheral nerve repair in human subjects. The animal models of stem-cell-induced peripheral nerve regeneration have shown impressive results; however, it is important to note that most of these models recreate acute nerve damage to mimic trauma and may recapitulate the same results in cases of chronic nerve damage such as neuropathy. Further experimentation is also necessary to better elucidate the long-term outcomes of stem cell therapy in peripheral nerve injury. Additionally, amidst its promising future, the use of any type of stem cell still has its ethical issues, which must be carefully considered before their introduction into the standard of care. Overall, it is evident that more clinical studies in human subjects must be conducted to standardize clinical protocols and portray an accurate representation of the scope of use for stem cell therapy in peripheral nerve injury. Future research should also be focused on combining stem cell lines to best recapitulate the intricate morphology of injured peripheral nerves. But ultimately, stem-cell-induced peripheral nerve regeneration appears to be a very viable therapeutic option for patients living with peripheral nerve damage.

## Figures and Tables

**Table 1:** Comparison between different cell types as sources for stem cells with advantages and disadvantages in relation to peripheral nerve regeneration (iPSCs: induced pluripotent stem cells, HFSCs: hair follicle stem cells, ESCs: embryogenic stem cells, ADSCs: adipose-derived stem cells, BMDSCs: bone-marrow-derived stem cells, SKMDSCs: skeletal-muscle-derived stem cells, hDPSCs: human dental pulp stem cells, NSCs: neural stem cells, EPCs: endothelial progenitor cells).

Cell Type	Advantages	Disadvantages	References
iPSCs	Autologous somatic cell use, avoidance of ethical concerns	Increased tumorgenicity, epigenetic memory	[25,26,27]
HFSCs	Lack of genetic manipulation needed, low tumorgenicity, can differentiate into SCs populations, ample source of extraction available	Time consuming and difficult isolation process	[26,28,29]
ESCs	Direct differentiation into somatic stem-cell-like precursors, abundant source of cells, long-term proliferation potential	Ethical concerns, teratoma and malignant teratocarcinoma formation	[25,30,31]
ADSCs	Secrete humoral factors that promote migration and proliferation of SCs, easy isolation, and accessibility	Preference for differentiation towards adipocytes	[8,26,32,33]
BMDSCs	Support nerve fiber regeneration, differentiate to Schwann-cell-like supportive cells	Painful, invasive extraction process, decreased proliferation and differentiation compared to other sources	[7,8,26]
SKMSCs	Multipotent differentiation potential	Minimal research available	[34]
hDPSCs	Easy extraction, neuroprotective and neurotrophic effects	Cryopreservation and storage requirements	[26,35,36]
NSCs	Autologous, same or similar cell origin as differentiation target	Harvest requires invasive brain surgery, difficult process to induce differentiation	[26,37]
EPCs	Simple to harvest from peripheral blood	Increased risk for malignant transformation, immunogenicity, or embolus formation	[26,34]

**Table 3:** Comparison and overview of the current significant findings of recently published or ongoing human clinical trials investigating the use of stem cell therapy in peripheral nerve regeneration and repair.

Clinical Trial	Type of Stem Cell Used	Nerve Targeted	Current Stage of Clinical Trial at Present	Trial Overview
BMAC Nerve Allograft Study	Autologous bone marrow aspirate concentrate	Peripheral Nerves	Finished	A prospective, phase I human safety study evaluating the consecutive treatments of the Avance Nerve Graft, a decellularized processed peripheral nerve allograft, with autologous bone marrow aspirate concentrate (BMAC). The purpose of this study was to evaluate the enrichment of regenerative ability by enhancing the scaffold with a patient’s own BMAC, and both produce a safety profile and provide proof of principle for the use of autologous stem cell transplants in conjunction with scaffolds [61].
Treatment of Optic Neuropathies Using Autologous Bone-Marrow-Derived Stem Cells	Bone-marrow-derived stem cells	Optic Nerve	Phase 2	This is an interventional, single-center trial created to evaluate the safety and efficacy of the use of purified adult autologous bone-marrow-derived CD34+, CD133+, and CD271+ stem cells to restore function of a damaged optic nerve through a 24-month follow-up period. The projected outcomes of this study are defined as a restoration of the functional capabilities of the damaged optic nerve, overall improvement of vision, and improvement in quality of life of patients [62].
Autologous Adipose Mesenchymal Stem Cell Transplantation in the Treatment of Patients with Hemifacial Spasm	Autologous adipose stem cells	Facial Nerve	Early Phase 1	This is an interventional study attempting to use adipose stem cell transplantation wrapped around an injured facial nerve to treat microvascular decompression hemifacial spasm in patients to improve nerve function, obtain better recovery, understand the efficacy of using stem cells in the treatment of cranial nerve dysfunctions, and provide evidence for the further treatment of other cranial nerve dysfunctions [63].
Human Amniotic Membrane and Mesenchymal Stem Cell Composite (BPI + MSC)	Adipose-derived mesenchymal stem cells	Brachial Plexus	Finished	This is a non-randomized, single-center clinical trial created to investigate the use of human amniotic membrane and allogeneic adipose-derived mesenchymal stem cells as a wrapping apparatus to enhance the nerve transfer process of upper traumatic brachial plexus injury, with a focus on the improvement of axonal regeneration [64].

## Data Availability

No new data were created or analyzed in this study. Data sharing does not apply to this article.
